# The Dynamic Immune Response of Yellow Catfish (*Pelteobagrus fulvidraco*) Infected With *Edwardsiella ictaluri* Presenting the Inflammation Process

**DOI:** 10.3389/fimmu.2021.625928

**Published:** 2021-02-26

**Authors:** Xu Zhou, Gui-Rong Zhang, Wei Ji, Ze-Chao Shi, Xu-Fa Ma, Zun-Lan Luo, Kai-Jian Wei

**Affiliations:** ^1^National Demonstration Center for Experimental Aquaculture Education, Huazhong Agricultural University, Wuhan, China; ^2^Key Laboratory of Freshwater Animal Breeding, Ministry of Agriculture and Rural Affairs, College of Fisheries, Huazhong Agricultural University, Wuhan, China; ^3^State Key Laboratory of Environmental Criteria and Risk Assessment, Chinese Research Academy of Environmental Sciences, Beijing, China; ^4^Key Laboratory of Freshwater Biodiversity Conservation, Ministry of Agriculture and Rural Affairs, Yangtze River Fisheries Research Institute, Chinese Academy of Fishery Sciences, Wuhan, China

**Keywords:** transcriptome, bacteria, *Edwardsiella ictaluri*, yellow catfish, inflammation

## Abstract

*Edwardsiella ictaluri* is a highly destructive pathogen in cultured yellow catfish, thus it was very necessary to study the immune response of yellow catfish against bacterial infection. In this study, RNA-Seq technology was used to study the immune response in two distinct tissues of yellow catfish at eight different time points (h) after *E. ictaluri* infection. The number of differentially expressed genes (DEGs) in the spleen and liver was low at 3 h and 6 h post-infection, respectively. Afterwards, the most number of DEGs in the spleen was detected at 72 h, while the number of DEGs in the liver maintained a high level from 24 h to 120 h. The GO and KEGG enrichment analyses of DEGs at different time points uncovered that cytokines were continuously transcribed at 6 h to 120 h; whereas the liver is the main organ that secretes the components of the complement system, and metabolic regulation was activated from 12 h to 120 h. Moreover, an overview of the inflammation response of yellow catfish was exhibited including pattern-recognition receptors, inflammatory cytokines, chemokines, complements, and inflammation-related signal pathways. The similar expression tendency of nine genes by qRT-PCR validated the accuracy of transcriptome analyses. The different transcriptomic profiles obtained from the spleen and liver will help to better understand the dynamic immune response of fish against bacterial infection, and will provide basic information for establishing effective measures to prevent and control diseases in fish.

## Introduction

RNA-Seq technology, as a powerful tool, has been widely exploited to study gene expression profiling in both physiological and pathological conditions (1, 2). In recent years, transcriptome analyses about immune response against bacterial infection have been performed in various fishes (3–22). Several pathogenic bacteria (e.g. *Aeromonas hydrophila*, *Edwardsiella tarda*, *Vibrio alginolyticus*) and some tissues (e.g. spleen, liver, head kidney) were commonly used as sources of infection and target tissues in fish transcriptome analyses, respectively (23). After bacterial infection, complement system, pattern recognition pathways, antigen processing and presentation pathway, and B cell and T cell receptor signaling pathway can be activated in various fishes (23). Though these studies have revealed the immune mechanism of fish underlying the bacteria-infection process, there has been limited information to use transcriptome analysis to study the dynamic immune process of fish in time and space.

Yellow catfish (Pelteobagrus fulvidraco), an important commercial freshwater species, is very popular as food in China, Japan, South Korea and Southeast Asia (24). With the increasing market demand in China, the aquaculture of yellow catfish has developed rapidly in recent years. However, bacterial diseases and parasitoses often outbreak and have caused great economic losses (25). Edwardsiella ictaluri, a Gram-negative rod-shaped bacterium, was reported to cause severe ascites disease, enteric septicemia and crack-head disease in yellow catfish (26, 27). In mammals, inflammation is a protective response of the organism when host cells sense evolutionarily conserved structures through germline-encoded pattern-recognition receptors (PRRs) (28). Once inflammatory processes are triggered, large amounts of pro-inflammatory cytokines are released, such as tumor necrosis factor (TNF), interleukin-1β (IL-1β), interferon-γ (IFN-γ), interleukin-17 (IL-17), and interleukin-22 (IL-22) (28). Chemokines are messengers of innate immunity, and they can recruit leukocytes into tissues at the site of infection to prevent and eliminate pathogens (29). Besides, the complement system is an important and efficient immune defense mechanism, which mediates microbial opsonization and killing, and generates inflammatory peptides such as C3a and C5a (28). In teleosts, the inflammatory mechanisms are not clear underlying the bacteria-infection process. Thus, it is necessary to understand the inflammatory process against bacterial infection and establish effective measures to prevent and control diseases in yellow catfish.

In recent years, there have been a few researches about the transcriptome of yellow catfish after stimulants and bacteria stimulation. A comparative transcriptome analysis between wild and albino yellow catfish provided important basic information for comparative genomics, evolution, and genetic breeding in this species (30). After stimulation by lipopolysaccharide (LPS) and polyriboinosinic polyribocytidylic acid (poly I:C) for 12 h, transcriptomic analysis of yellow catfish liver observed 370 differentially expressed genes (DEGs) including 18 immune response genes, and 522 DEGs including 13 immune response genes, respectively (31, 32). Transcriptome profile of yellow catfish spleen following *E. ictaluri* challenge for 72 h detected 172 immune DEGs that were enriched into the immune response-related pathways (pattern recognition receptor, complement and coagulation cascades, T-cell receptor and B-cell receptor signaling pathways) (22). Although the above transcriptome studies analyzed the immune response and potential molecular mechanism of yellow catfish against bacterial infection based on a single tissue at one treatment time point, it is necessary to further investigate the dynamic immune response of yellow catfish against bacteria based on several tissues at different time points post-infection. The spleen is an important immune organ that not only has a large number of macrophages and neutrophils but also can generate a lot of cytokines and chemokines against pathogenic infection (33). The liver links host defense with metabolic readjustments and plays a dual role in immune function and metabolism upon pathogen challenge (34). In this study, the transcriptomic profiles were obtained by RNA-Seq technology from the spleen and liver tissues of yellow catfish at 0 h (control), and 3 h, 6 h, 12 h, 24 h, 48 h, 72 h, and 120 h after infection with *E. ictaluri*. This study aims to understand the dynamic immune response of yellow catfish against bacterial infection and to explore the process of the inflammatory response against bacterial infection.

## Materials and Methods

### Fish Collection

Healthy juvenile individuals of yellow catfish (one-year-old, ~14 g) were obtained from the fish breeding base of Huazhong Agricultural University (HZAU). Before bacterial infection, the fishes were acclimatized to laboratory conditions in two circulating water tanks by keeping the temperature at 27±1 °C and were fed a commercial diet (Hubei Haid Feeds Company, Wuhan, China) twice a day (09:00 and 16:00).

### Experimental Infections and Sampling Procedures

The bacteria (*E. ictaluri*) for immune challenge experiments were obtained from the fish immunology laboratory of HZAU, and were cultured on brain–heart infusion (BHI) (Becton, Dickinson and Company, USA) and incubated 12 h at 28 °C in the incubator. Subsequently the bacteria were washed and suspended in phosphate-buffered saline (PBS, pH 7.2) to a final concentration of 5×10^6^ CFU/mL. Each fish in the experimental group was intraperitoneally injected with 50 μL of suspended *E. ictaluri* (i.e. 2.5×10^5^ CFU/fish) and each fish in the control group was injected with the same volume of PBS. Twelve fishes were randomly sampled from the experimental group at 3, 6, 12, 24, 48, 72, and 120 h post-injection of *E. ictaluri* and from the control group at 0 hours, every four fishes were as a group of biological duplication. The sampled fish were euthanatized with 300 mg/L MS-222, and then the spleen and liver tissues were collected for RNA extraction. All tissues were immediately frozen in liquid nitrogen and stored at −80 °C until RNA extraction. All infection experiments and sample collections adhered to the standard biosecurity and institutional safety procedures of HZAU.

### RNA Extraction and cDNA Synthesis

Total RNA was extracted from various tissues using Trizol Reagent (Invitrogen, USA) according to the manufacturer’s instruction. The quality of total RNA was checked by 1% agarose gel electrophoresis. The concentration of total RNA was determined using a Nanodrop ND-2000 spectrophotometer (Thermo Electron Corporation, USA). The first-strand cDNA was generated using the Revert Aid™ M-MLV Reverse Transcriptase Kit (Promega, USA) following the manufacturer’s instructions. The cDNA products were stored at −20 °C.

### RNA Sequencing and Mapping Procedures

RNA samples were packed in dry ice and shipped to the Majorbio Biotech Co., Ltd. (Shanghai, China) for further analysis. The RNA-seq libraries were prepared using protocols supplied with the TruSeq™ RNA sample preparation Kit (Illumina, San Diego, CA, USA). FASTX-Toolkit (http://hannonlab.cshl.edu/fastx_toolkit/) was used to evaluate the quality of every sample. SeqPrep (https://github.com/jstjohn/SeqPrep) and Sickle (https://github.com/najoshi/sickle) were used to filter out reads with adaptors and low-quality reads from raw data to generate clean data. The clean reads were assembled using Trinity (https://github.com/trinityrnaseq/trinityrnaseq). High-quality RNA sequences were obtained after filtration by TransRate (http://hibberdlab.com/transrate/) and CD-HIT (http://weizhongli-lab.org/cd-hit/). The assembled integrity of transcriptome was assessed by BUSCO (http://busco.ezlab.org). High-quality RNA-Seq reads were mapped with the clean reads.

### Identification of Differentially Expressed Genes

To identify differentially expressed genes (DEGs), cleaned, mapped and counted datasets obtained from experimental groups were compared to the control group (0 h). Only the genes with more than 10 reads in at least one of the individual libraries were applied to DEGs analyses using DESeq2 package. Finally, DEGs with adjusted *P*≤0.05 and |log_2_(fold-change)| ≥1 were considered as the targets for further analyses. The data of transcriptome were annotated using GO (BLAST2GO) and KEGG (KOBAS) database. The enrichment analyses of GO and KEGG were performed using Goatools and Python softwares, respectively.

### Bioinformatic Analysis

The complete sequence of the open reading frame (ORF) of each target gene was found using ORF Finder (http://www.ncbi.nlm.nih.gov/projects/gorf/). The protein structure of each target gene was predicted by Simple Modular Architecture Research Tool (SMART) (http://smart.embl-heidelberg.de/). ClustalW program in MEGA 6.06 and BoxShade (http://www.ch.embnet.org/software/BOX_form.html) was used for multiple sequence alignments.

### Analysis of Gene Expression by Quantitative Real-Time PCR (qRT-PCR)

qRT-PCR was used to detect mRNA expression levels of selected DEGs in the spleen and liver using a 6300 RT-PCR system (Applied Biosystems, USA). The gene-specific primer pairs for qRT-PCR were designed based on the RNA sequences of selected DEGs (**Supplemental Table 1**). The β-actin gene was used as an internal control gene. The PCR reaction mixture consisted of 10 μL LightCycler® 480 SYBR Green I Master (Roche, Germany), 7 μL ddH_2_O, 2 μL cDNA (3 times dilution of a template) and 0.5 μL of either gene-specific primer (10 μM) in a total volume of 20 μL. The qRT-PCR of each sample was performed in triplicate according to the following conditions: 95 °C for 10 min, followed by 40 cycles at 95 °C for 15 s, annealing temperature for 30 s and 72 °C for 30 s. At the end of each PCR reaction, amplification curve and melting curve analyses were performed to check the integrity of the reaction and the quality of the product, respectively. To compare expressions of selected DEGs, the 2^-ΔΔCt^ method was adopted to calculate the relative expression levels of the target genes (35).

## Results

### Transcriptomic Sequencing and Raw Reads Data

Forty-two high-quality cDNA libraries from the liver and spleen were constructed by Illumina Hiseq 2000 sequencing platform (**Supplemental Table 2**). In the liver samples, the raw reads obtained were between 41,039,960 and 75,135,220. The Q20 and Q30 values of these reads were more than 98 and 95%, respectively, the error rates were less than 0.024%, and the CG contents were about 48%. In the spleen samples, the raw reads ranged from 44,062,228 to 62,082,818. The Q20 and Q30 quality scores of all reads were more than 97 and 93%, respectively, the error rates were less than 0.027%, and the CG contents were between 45.02 and 52.67% (**Table 1**). After filtration, the clean reads were mapped back to yellow catfish sequences, and the mapped rate of each sample was over 82% in the liver samples and over 75% in the spleen samples (**Supplemental Table 3**).

**Table 1:** Statistics of differentially expressed genes (DEGs) at different time points following *E. ictaluri* challenge.

Time	Spleen			Liver		
	Up-regulated DEGs (%)	Down-regulated DEGs (%)	Total	Up-regulated DEGs (%)	Down-regulated DEGs (%)	Total
3h	133 (85.26)	23 (14.74)	156	318 (79.70)	81 (20.30)	399
6h	485 (90.99)	48 (9.01)	533	335 (74.78)	113 (25.22)	448
12h	1,192 (86.06)	193 (13.94)	1,385	1,025 (70.74)	424 (29.26)	1,449
24h	1,906 (74.71)	645 (25.29)	2,551	1,630 (56.05)	1,278 (43.95)	2,908
48h	1,621 (68.54)	744 (31.46)	2,365	1,740 (55.27)	1,408 (44.73)	3,148
72h	2,648 (62.15)	1,612 (37.85)	4,260	1,725 (55.97)	1,357 (44.03)	3,082
120h	2,156 (66.56)	1,083 (33.44)	3,239	1,594 (53.17)	1,404 (46.83)	2,998
Total	3,630	1,924	5,540	2,364	1,943	4,161

### Analysis of Differentially Expressed Genes (DEGs)

All the clean reads were assembled into 481,943 transcripts with a min length of 201 bp, a max length of 10,109 bp and an N50 length of 1,368 bp, and 321,257 unigenes were further assembled (**Supplemental Table 4**). By comparing the unigenes from the experimental groups with that from the control group based on the criteria for DEGs, we screened 5,540 and 4,161 DEGs in the spleen and liver, respectively (**Table 1**).

In the spleen samples, the total numbers of up- and down-regulated DEGs were 3,630 and 1,924, respectively (**Table 1**). The total number of DEGs was lower at 3 h and 6 h post-infection than that at 12–120 h, and the number of up-regulated DEGs was significantly higher than that of down-regulated DEGs at each time point. Besides, the number of up- and down-regulated DEGs in the spleen was continuously increased and reached the highest level at 72 h post-infection, except for a slight decline of the up-regulated DEGs at 48 h.

In the liver samples, the total numbers of up- and down-regulated DEGs were 2,364 and 1,943, respectively (**Table 1**). The total number of DEGs was also fewer at 3 h and 6 h than that at other time points. Furthermore, the proportion of up-regulated DEGs (70.74–79.70%) was significantly higher than that of down-regulated DEGs (20.30–29.26%) at 3 h, 6 h, and 12 h, while the proportion of up-regulated DEGs (53.17–56.05%) was slightly higher than that of down-regulated DEGs (43.95–46.83%) at 24–120 h. The number of up- and down-regulated DEGs in the liver gradually increased to a high level at 24 h, and then hit a peak at 48 h.

### Function Analysis of Differentially Expressed Genes (DEGs)

In total, 3,335 GO annotated DEGs and 943 KEGG annotated DEGs were detected in the spleen, whilst a total of 2,851 and 975 DEGs were annotated by the GO and KEGG database of the liver, respectively (**Table 2**). Of the total DEGs in the spleen and liver at different time point post-infection, the proportion of GO annotated DEGs was distinctly higher than that of KEGG annotated DEGs.

**Table 2:** Annotated DEGs in gene ontology (GO) and KEGG database.

Time	Spleen			Liver		
	Total DEGs	GO Annotated DEGs (%)	KEGG Annotated DEGs (%)	Total DEGs	GO Annotated DEGs (%)	KEGG Annotated DEGs (%)
3h	156	109 (69.87)	53 (33.97)	399	213 (53.38)	66 (16.54)
6h	533	327 (61.35)	144 (27.01)	448	278 (62.05)	74 (16.52)
12h	1385	863 (62.31)	213 (15.38)	1,449	929 (64.11)	328 (22.64)
24h	2,551	1,649 (64.64)	533 (20.89)	2,908	2,005 (68.95)	659 (22.66)
48h	2,365	1,529 (64.65)	319 (13.49)	3,148	2,199 (69.85)	715 (22.71)
72h	4,260	2,632 (61.78)	592 (13.90)	3,082	2,130 (69.11)	689 (22.36)
120h	3,239	2,044 (63.11)	526 (16.24)	2,998	2,099 (70.01)	731 (24.38)
Total	5,540	3,335 (60.20)	943 (17.02)	4,161	2,851 (68.52)	975 (23.43)

**Figure 1:**
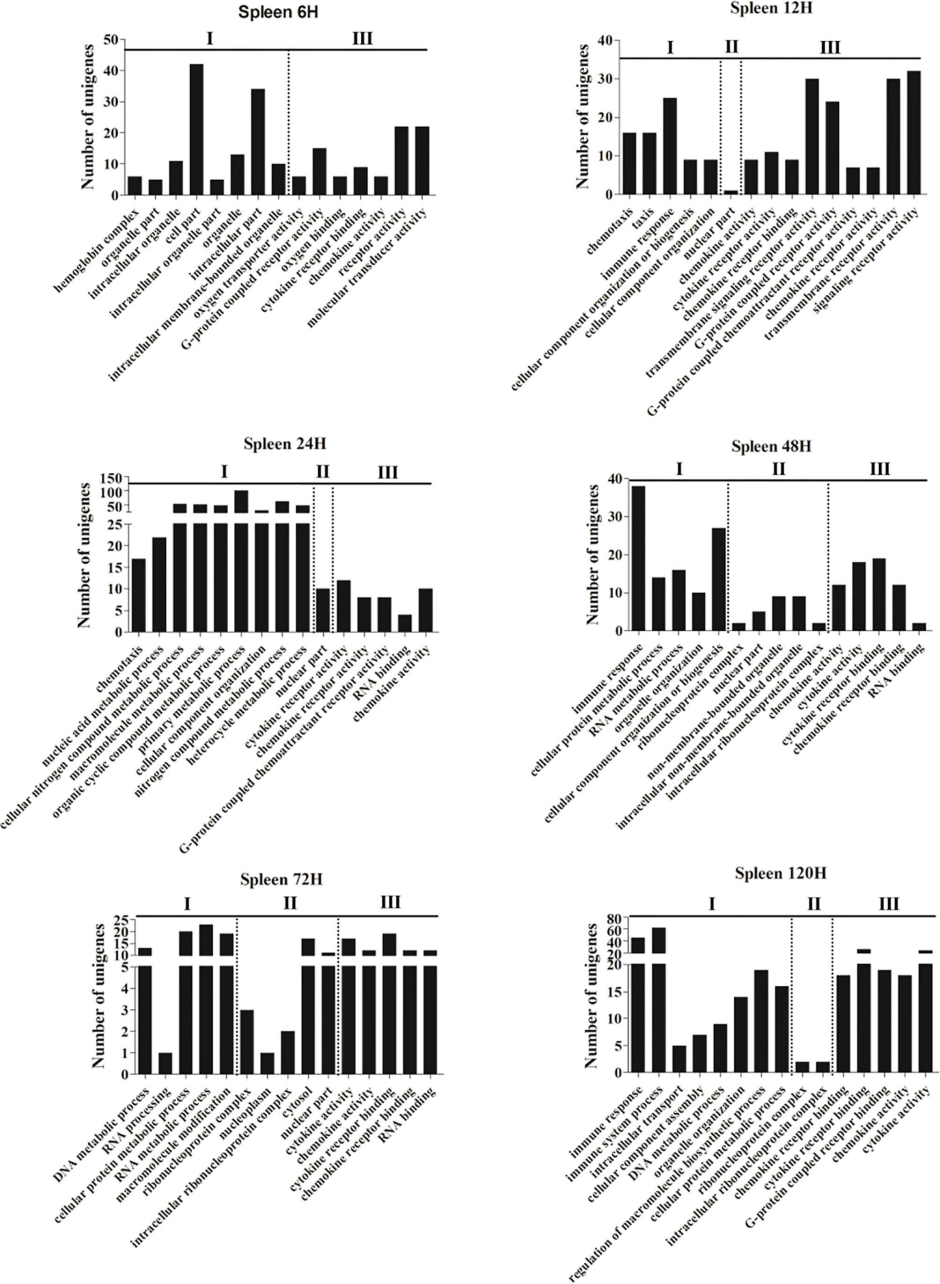
The 15 most enriched GO terms in the spleen of yellow catfish at different time points following *E. ictaluri* challenge. I: biological process, II: cellular component, III: molecular function.

**Figure 2:**
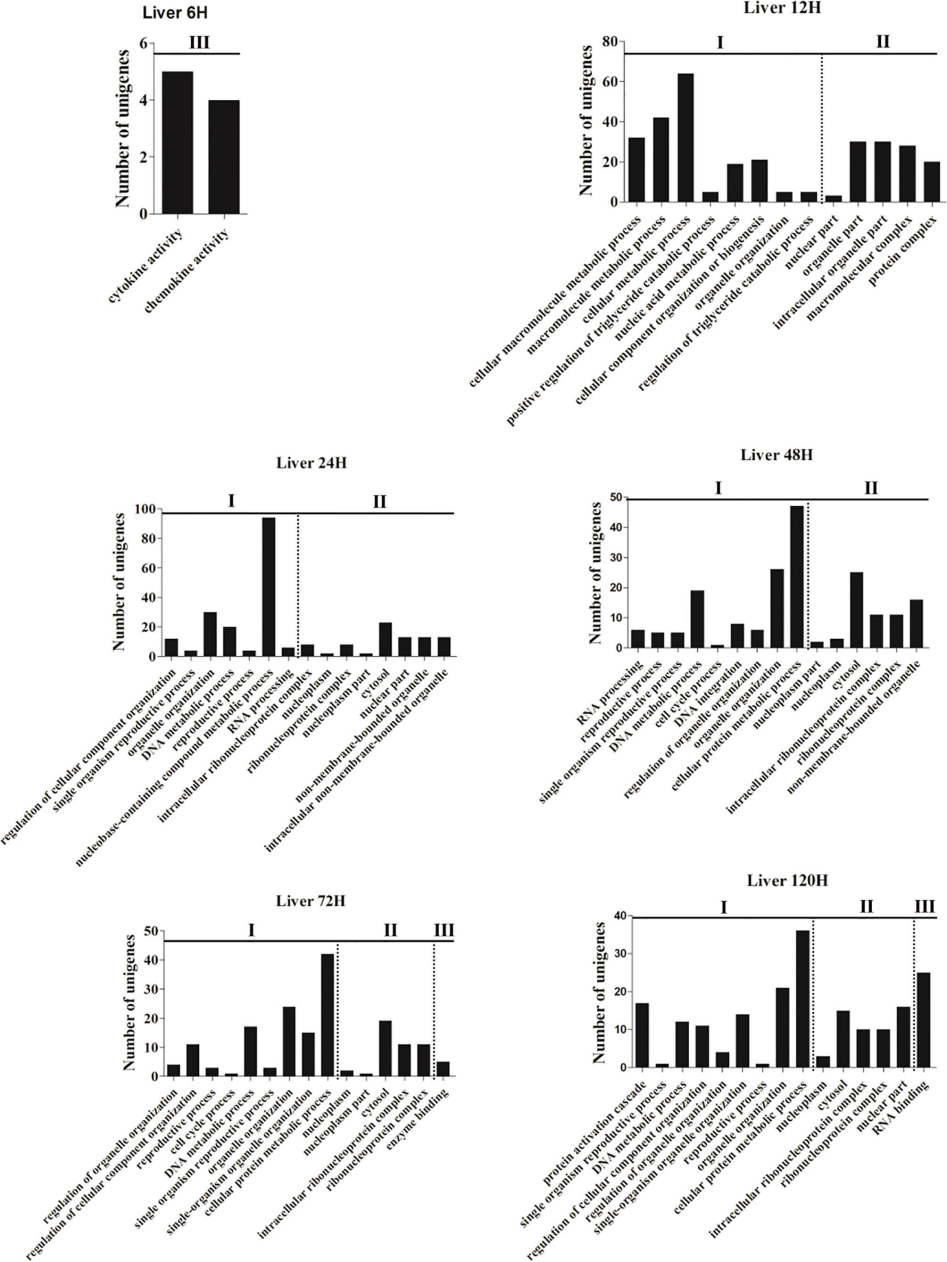
The 15 most enriched GO terms in the liver of yellow catfish at different time points following *E. ictaluri* challenge. I: biological process, II: cellular component, III: molecular function.

To investigate the dynamic change of the immune functions at different time points post-infection, GO and KEGG enrichments were performed based on the DEGs with *P*-value <0.05 and |log_2_(fold-change)| ≥2 from the spleen and liver, respectively. Fifteen most enriched GO terms (*P*<0.01) of three subclasses (biological process, cellular component and molecular function) were selected in the spleen and liver at 6–120 h post-infection, respectively (**Figures 1** and **2**). No GO terms were enriched at 3 h in both spleen and liver. In the spleen, the majority of GO terms were distributed in the biological processes and molecular functions except for the 48 h and 72 h time points (**Figure 1**). In the molecular functions, cytokine and chemokine associated GOs were detected from 6 h to 120 h, G-protein receptor-associated GOs were found at 6 h, 12 h, 24 h, and 120 h. Moreover, in the biological processes, immune response-associated GOs were detected at 12 h, 48 h, and 120 h, chemotaxis was found at 12–24 h, metabolic process associated GOs were found at 24–120 h (**Figure 1**). In the liver, the majority of GOs were distributed in the biological processes and cellular components (**Figure 2**). Only cytokine activity and chemokine activity were discovered at 6 h. Metabolic process and regulation associated GOs were found at 12–120 h, and reproductive process associated GOs were detected at 24–120 h in the biological processes. In addition, nucleoplasm, intracellular ribonucleoprotein complex, ribonucleoprotein complex and cytosol were detected at 24–120 h in the cellular component (**Figure 2**).

According to KEGG enrichment analysis, ten most enriched pathways (*P*<0.01) were obtained in the spleen and liver at 6–120 h post-infection, respectively (**Tables 3** and **4**). No pathways were enriched at 3 h in both spleen and liver. In the spleen, hematopoietic cell lineage (map04640) and cytokine-cytokine receptor interaction (map04060) were detected at 6–120 h, chemokine signaling pathway (map04062) was detected at 12–120 h, TNF signaling pathway (map04668) was discovered at 6–120 h except for 48 h, complement and coagulation cascades (map04610) was detected at 12 h, 72 h, and 120 h, and IL-17 signaling pathway (map04657) was only found at 6 h and 12 h (**Table 3**). In the liver, complement and coagulation cascades (map04610) was detected at 24–120 h, TNF signaling pathway (map04668) was only found at 6 h and 12 h, IL-17 signaling pathway (map04657) was only detected at 6 h, and Th17 cell differentiation was only found at 12 h (**Table 4**). Besides, fat digestion and absorption (map04975) was found at 12–120 h, and PPAR signaling pathway (map03320) was enriched notably at 24–120 h (**Table 5**).

**Table 3:** The top 10 enriched KEGG pathways in the spleen at different time points following *E. ictaluri* challenge.

Time	Ko id	Term	Number of DEGs	*P* value
6h	map04640	Hematopoietic cell lineage	8	2.13E-07
	map04060	Cytokine-cytokine receptor interaction	9	6.33E-06
	map05144	Malaria	6	3.96E-05
	map00524	Neomycin, kanamycin and gentamicin biosynthesis	3	0.003885
	map04668	TNF signaling pathway	6	0.005152
	map05140	Leishmaniasis	6	0.005512
	map04657	IL-17 signaling pathway	6	0.005737
12h	map04060	Cytokine-cytokine receptor interaction	18	0
	map04062	Chemokine signaling pathway	17	1.95E-08
	map04640	Hematopoietic cell lineage	9	1.4E-06
	map04668	TNF signaling pathway	9	0.000269
	map05140	Leishmaniasis	9	0.000297
	map05120	Epithelial cell signaling in Helicobacter pylori infection	9	0.001552
	map05323	Rheumatoid arthritis	8	0.001825
	map04610	Complement and coagulation cascades	8	0.002612
	map04657	IL-17 signaling pathway	8	0.002848
	map04064	NF-kappa B signaling pathway	8	0.004825
24h	map04060	Cytokine-cytokine receptor interaction	29	0
	map04145	Phagosome	31	0
	map05323	Rheumatoid arthritis	21	0
	map05120	Epithelial cell signaling in Helicobacter pylori infection	20	6.58E-09
	map05110	Vibrio cholerae infection	18	2.82E-08
	map04062	Chemokine signaling pathway	23	3.73E-08
	map04640	Hematopoietic cell lineage	12	5.91E-07
	map04668	TNF signaling pathway	15	1.37E-06
	map05152	Tuberculosis	22	5.18E-06
	map04966	Collecting duct acid secretion	11	5.18E-06
48h	map04640	Hematopoietic cell lineage	14	0
	map04060	Cytokine-cytokine receptor interaction	34	0
	map04145	Phagosome	32	0
	map05323	Rheumatoid arthritis	23	0
	map04062	Chemokine signaling pathway	23	1.32E-07
	map05120	Epithelial cell signaling in Helicobacter pylori infection	18	3.11E-07
	map05110	Vibrio cholerae infection	16	9.58E-06
	map05152	Tuberculosis	22	2.84E-05
	map05164	Influenza A	21	8.45E-05
	map04966	Collecting duct acid secretion	10	0.000132
72h	map05323	Rheumatoid arthritis	23	0
	map04640	Hematopoietic cell lineage	17	0
	map04060	Cytokine-cytokine receptor interaction	33	0
	map04145	Phagosome	40	0
	map04062	Chemokine signaling pathway	27	8.27E-08
	map05205	Proteoglycans in cancer	32	2.94E-06
	map05110	Vibrio cholerae infection	17	9.61E-05
	map04933	AGE-RAGE signaling pathway in diabetic complications	18	0.000199
	map04610	Complement and coagulation cascades	15	0.000232
	map04210	Apoptosis	22	0.000300
120h	map04060	Cytokine-cytokine receptor interaction	35	0
	map05323	Rheumatoid arthritis	26	0
	map04610	Complement and coagulation cascades	19	4.79E-09
	map04145	Phagosome	28	8.3E-08
	map04640	Hematopoietic cell lineage	12	3.56E-06
	map04062	Chemokine signaling pathway	21	1.15E-05
	map05120	Epithelial cell signaling in Helicobacter pylori infection	16	3.4E-05
	map04933	AGE-RAGE signaling pathway in diabetic complications	17	3.92E-05
	map04668	TNF signaling pathway	14	7.61E-05
	map05150	Staphylococcus aureus infection	10	0.000269

**Table 4:** The top 10 enriched KEGG pathways in the liver at different time points following *E. ictaluri* challenge.

Time	Ko id	Term	Number of DEGs	*P* value
6 h	map04657	IL-17 signaling pathway	7	3.78E-07
	map04668	TNF signaling pathway	5	0.000332
	map05134	Legionellosis	5	0.001277
12 h	map04380	Osteoclast differentiation	15	2.54E-08
	map04668	TNF signaling pathway	13	4.96E-07
	map05162	Measles	16	1.71E-06
	map04141	Protein processing in endoplasmic reticulum	23	1.23E-05
	map05164	Influenza A	17	1.61E-05
	map04010	MAPK signaling pathway	19	1.99E-05
	map04975	Fat digestion and absorption	8	2.98E-05
	map05140	Leishmaniasis	11	5.75E-05
	map05134	Legionellosis	12	0.000112
	map04659	Th17 cell differentiation	11	0.000377
24 h	map03320	PPAR signaling pathway	26	0
	map04141	Protein processing in endoplasmic reticulum	51	0
	map04975	Fat digestion and absorption	14	8.89E-07
	map04610	Complement and coagulation cascades	19	3.31E-05
	map05140	Leishmaniasis	18	0.000166
	map00561	Glycerolipid metabolism	18	0.000965
	map04933	AGE-RAGE signaling pathway in diabetic complications	20	0.001435
	map05134	Legionellosis	19	0.001854
	map04920	Adipocytokine signaling pathway	16	0.005000
	map03060	Protein export	13	0.007140
48 h	map04610	Complement and coagulation cascades	40	0
	map04975	Fat digestion and absorption	16	3.21E-08
	map03320	PPAR signaling pathway	26	2.38E-07
	map05150	Staphylococcus aureus infection	17	4.08E-07
	map00340	Histidine metabolism	15	4.16E-06
	map00140	Steroid hormone biosynthesis	13	3.96E-05
	map04933	AGE-RAGE signaling pathway in diabetic complications	24	6.65E-05
	map00380	Tryptophan metabolism	18	0.000127
	map05140	Leishmaniasis	18	0.001038
	map00561	Glycerolipid metabolism	19	0.001551
72 h	map04610	Complement and coagulation cascades	32	1.17E-08
	map04975	Fat digestion and absorption	16	1.42E-08
	map05150	Staphylococcus aureus infection	17	8.68E-08
	map03320	PPAR signaling pathway	22	1.54E-05
	map05140	Leishmaniasis	19	5.25E-05
	map04141	Protein processing in endoplasmic reticulum	41	0.001184
	map00561	Glycerolipid metabolism	18	0.001366
	map05142	Chagas disease (American trypanosomiasis)	21	0.001662
	map00340	Histidine metabolism	11	0.002574
	map05020	Prion diseases	11	0.003777
120 h	map05150	Staphylococcus aureus infection	18	0
	map04610	Complement and coagulation cascades	33	0
	map04975	Fat digestion and absorption	14	3.66E-07
	map05140	Leishmaniasis	21	4.22E-07
	map03320	PPAR signaling pathway	22	3.2E-06
	map04933	AGE-RAGE signaling pathway in diabetic complications	22	3.51E-05
	map00140	Steroid hormone biosynthesis	11	0.000285
	map05322	Systemic lupus erythematosus	16	0.000407
	map04974	Protein digestion and absorption	21	0.000445
	map05146	Amoebiasis	20	0.000559

**Table 5:** The only 10 inflammation-related signal pathways from all DEGs by KEGG enriched analysis.

Ko id	Term	Number of DEGs	*P* value
map04610	Complement and coagulation cascades	122	0
map04010	MAPK signaling pathway	117	4.09E-06
map04621	NOD-like receptor signaling pathway	95	3.38E-05
map04062	Chemokine signaling pathway	93	2.25E-08
map04060	Cytokine-cytokine receptor interaction	80	0
map04670	Leukocyte transendothelial migration	67	0.000828
map04659	Th17 cell differentiation	64	1.35E-07
map04612	Antigen processing and presentation	60	1.17E-05
map04630	JAK-STAT signaling pathway	56	1.89E-05
map04668	TNF signaling pathway	52	5.77E-06

### Inflammation-Related Genes of Yellow Catfish Against *E. ictaluri* Infection

**Figure 3:**
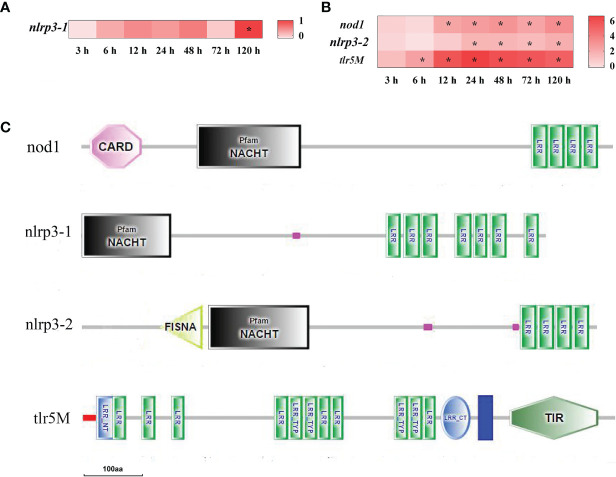
The DEGs of pattern-recognition receptor in yellow catfish after *E. ictaluri* infection. **(A**, **B)**: The DEGs identified in the spleen and liver, respectively. The DEGs of pattern-recognition receptor were analyzed at 3 h, 6 h, 12 h, 24 h, 48 h, 72 h, and 120 h post-infection in the spleen and liver. The color gradient represents highly up-regulated (red) to highly down-regulated (white) genes. Significant differences at different time points post-infection compared to the control (0 h) are indicated by asterisks (*P < 0.05). **(C)**: Structural features of pattern-recognition receptor DEGs in yellow catfish. The domain organizations were predicted by the SMART online server.

To analyze the dynamic inflammatory response of yellow catfish against bacterial infection, inflammation-related DEGs were screened in the spleen and liver at different time points after infection of *E. ictaluri*. The results showed that there were only four DEGs of pattern-recognition receptor found in the spleen and liver after infection (**Figure 3**). In the spleen, only NACHT, LRR, and PYD domains-containing protein 3 (nlrp3-1) mRNA was significantly up-regulated at 120 h (*P*<0.05) (**Figure 3A**). In the liver, Toll-like receptor 5 membrane (tlr5M), nucleotide-binding oligomerization domain-containing protein 1 (nod1) and nlrp3-2 mRNA were significantly up-regulated at 6–120 h, 12–120 h, and 24–120 h, respectively (*P*<0.05) (**Figure 3B**). The structure predictions of DEGs showed that the nod1, nlrp3-1 and nlrp3-2 all contained an NAIP, CIIA, HET-E, and TP1 (NACHT) nucleotide binding domain and a leucine-rich repeat (LRR) domain, while the nod1 also had a C-terminal caspase recruitment domain (CARD), and the nlrp3-2 also had a fish-specific NACHT associated domain (FISNA). The tlr5M contained a signal peptide, twelve LRR domains, an LRR C-terminal domain, a transmembrane domain and a TIR domain (**Figure 3C**).

**Figure 4:**
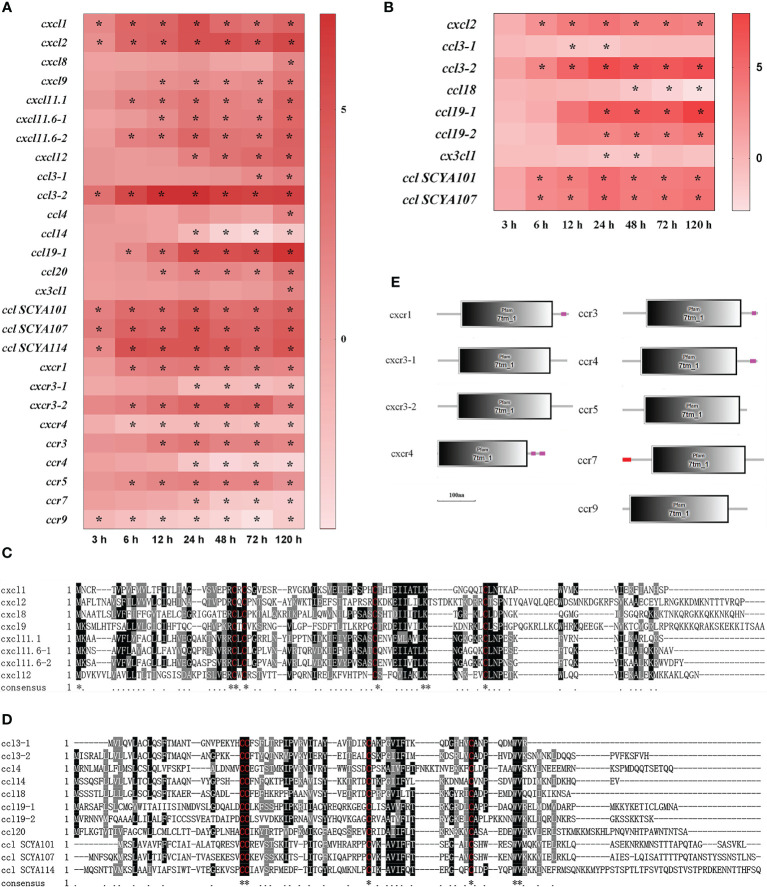
The DEGs of chemokine in yellow catfish after *E. ictaluri* infection. **(A**, **B)**: The DEGs identified in the spleen and liver, respectively. The DEGs of chemokine were analyzed at 3 h, 6 h, 12 h, 24 h, 48 h, 72 h, and 120 h post-infection in the spleen and liver. The color gradient represents highly up-regulated (red) to highly down-regulated (white) genes. Significant differences at different time points post-infection compared to the control (0 h) are indicated by asterisks (*P < 0.05). **(C**, **D)**: Multiple alignments of the deduced amino acid sequences of detected CXCLs and CCLs, respectively. The amino acid sequences of these genes were predicted using MEGA 6.06. ClustalW program in MEGA 6.06 and BoxShade were used for multiple sequence alignments. Similar amino acid residues are marked as grey shadow and identical residues as black shadow. Absent amino acids are indicated by dashes (-). **(E)**: Structural features of chemokine receptor DEGs in yellow catfish. The domain organizations were predicted by the SMART online server.

In total, 29 DEGs of chemokine and chemokine receptor were found in the spleen and liver after infection (**Figure 4**). Firstly, eight CXC chemokines (cxcl) were detected in the spleen and liver (**Figure 4**). In the spleen, cxcl1 and cxcl2 mRNAs were notably up-regulated at 3–120 h (*P*<0.05), cxcl8 mRNA was only significantly up-regulated at 120 h, whereas other five cxcls (cxcl9, cxcl11.1, cxcl11.6-1, cxcl11.6-2, and cxcl12) mRNAs were significantly up-regulated from 6 h (or 12 h, 48 h) to 120 h (*P*<0.05) (**Figure 4A**). In the liver, however, only cxcl2 mRNA was detected to be up-regulated significantly at 6–120 h (*P*<0.05) (**Figure 4B**). Secondly, a total of eleven CC chemokines (ccl) were found in the spleen and liver (**Figure 4**). In the spleen, ccl3-2, ccl SCYA 101, ccl SCYA 107, and ccl SCYA 114 mRNAs were significantly up-regulated at 3–120 h, ccl19-1 and ccl20 mRNAs were significantly up-regulated from 6 h and 12 h to 120 h, and ccl3-1 and ccl4 mRNAs only had high expression levels at 72–120 h and at 120 h, respectively (*P*<0.05), whereas ccl14 mRNA was notably down-regulated at 24–120 h (*P*<0.05) (**Figure 4A**). In the liver, ccl3-2, ccl SCYA 101 and ccl SCYA 107 mRNAs were significantly up-regulated at 6–120 h, ccl19-1, and ccl19-2 mRNA had high expression levels at 24–120 h (*P*<0.05), whereas ccl3-1 and ccl18 mRNAs were notably down-regulated at 12–24 h and at 48–120 h, respectively (*P*<0.05) (**Figure 4B**). Thirdly, four CXC chemokine receptors (cxcr) were found in the spleen, cxcr1 and cxcr3-2 mRNAs were notably up-regulated at 6–120 h, whereas cxcr4 and cxcr3-1 mRNAs were significantly down-regulated at 6–120 h and at 24–120 h, respectively (*P*<0.05) (**Figure 4A**). Finally, five CC chemokine receptors (ccr) detected in the spleen, of which ccr3 and ccr5 mRNAs were notably up-regulated from 6 h and 12 h to 120 h, respectively (*P*<0.05), whereas ccr4 and ccr7 mRNAs were significantly down-regulated at 24–120 h (*P*<0.05), and ccr9 mRNA was significantly down-regulated at 3–120 h (*P*<0.05) (**Figure 4A**). The alignment of protein sequences showed that all chemokines contained four conserved cysteines, two of which near C-terminal formed C-X-C structures called CXCL, or formed conserved C-C structures called CCL (**Figures 4C, D**). Moreover, the structure predictions showed that all chemokine receptors had a seven-transmembrane (7tm) domain (**Figure 4E**).

**Figure 5:**
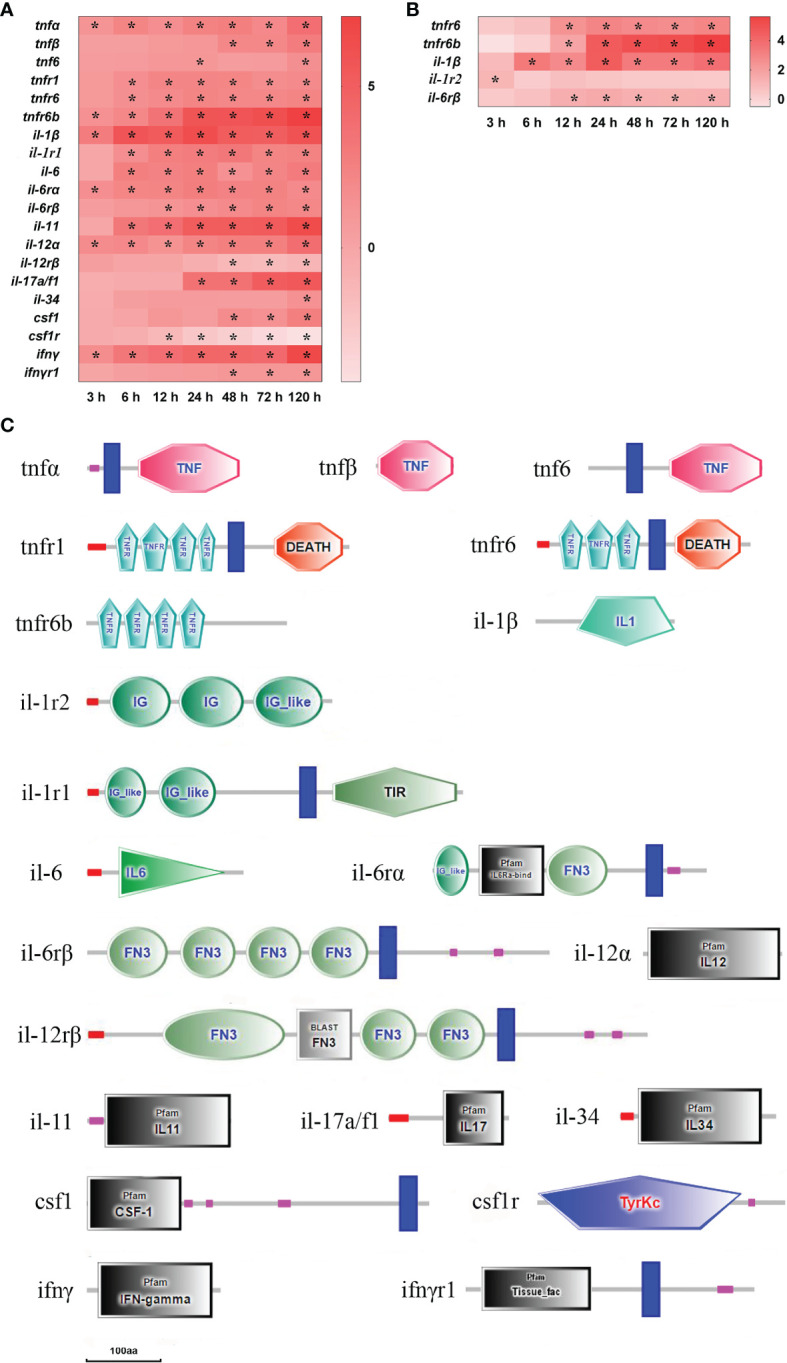
The DEGs of inflammatory cytokine in yellow catfish after *E. ictaluri* infection. **(A**, **B)**: The DEGs identified in the spleen and liver, respectively. The DEGs of inflammatory cytokine were analyzed at 3 h, 6 h, 12 h, 24 h, 48 h, 72 h and 120 h post-infection in the spleen and liver. The color gradient represents highly up-regulated (red) to highly down-regulated (white) genes. Significant differences at different time points post-infection compared to the control (0 h) are indicated by asterisks (*P < 0.05). **(C)**: Structural features of inflammatory cytokine DEGs in yellow catfish. The domain organizations were predicted by the SMART online server.

After infection with *E. ictaluri*, 21 DEGs of inflammatory cytokine and inflammatory cytokine receptor were found in the spleen and liver (**Figure 5**). Firstly, there were three tumor necrosis factors (tnf) and three tnf receptors (tnfr) detected in the spleen and liver. In the spleen, tnfα and tnfr6b mRNAs were significantly up-regulated at 3–120 h (*P*<0.05), and tnfr1 and tnfr6 mRNAs were significantly up-regulated at 6–120 h, while tnfβ and tnf6 mRNAs were notably up-regulated at 48–120 h and at 24 h/120 h, respectively (*P*<0.05) (**Figure 5A**). In the liver, only tnfr6 and tnfr6b mRNAs were significantly up-regulated at 12–120 h (*P*<0.05) (**Figure 5B**). The structure predictions of DEGs showed that the tnfα, tnfβ and tnf6 had a typical TNF family domain, the tnfr1 contained a signal peptide, four TNFR family domains, a transmembrane region and a death domain, whereas the tnfr6 only had three TNFR domains compared with the tnfr1 (**Figure 5C**). Secondly, there were six interleukins (il) and five interleukin receptors (ilr) found in the spleen and liver. In the spleen, il-1β, il-12α and il-6rα mRNAs were notably up-regulated at 3–120 h, il-6, il-11, and il-1rI;-1 mRNAs were markedly up-regulated at 6–120 h, il-6rβ, and il-17a/f1 mRNAs were significantly up-regulated from 12 h and 24 h to 120 h, respectively (*P*<0.05), whereas il-12rβ mRNA was significantly down-regulated at 48–120 h (*P*<0.05) and il-34 mRNA was notably up-regulated only at 120 h (*P*<0.05) (**Figure 5A**). In the liver, il-1β, il-6rβ, and il-1rI;-2 mRNAs had high expression levels at 6–120 h, 12–120 h, and 3h, respectively (*P*<0.05) (**Figure 5B**). Thirdly, colony stimulating factor 1 (csf1), csf1receptor (csf1r), interferon γ (ifnγ), and ifnγ receptor (ifnγr) were only detected in the spleen. The ifnγ mRNA was significantly up-regulated at 3–120 h with ifnγr mRNA up-regulated at 48–120 h (*P*<0.05). The csf1 mRNA was significantly up-regulated at 48–120 h, whereas csf1r mRNA was notably down-regulated at 12–120 h (*P*<0.05) (**Figure 5A**). The structure predictions of DEGs showed that the il-1β had a typical IL-1 family domain, the il-1 receptor type I; (il-1r1) consisted of a signal peptide, two IG-like domains, a transmembrane region and a TIR domain, and the il-1 receptor type II; (il-1r2) contained two IG domains and an IG-like domain (**Figure 5C**). The il-6 contained a conserved IL-6 family domain; the il-6rα had an IL-6ra-bind domain, a fibronectin type 3 (FN3) domain and a transmembrane region, and the il-6rβ comprised four FN3 domains and a transmembrane region (**Figure 5C**). The il-12α had a typical IL-12 family domain, and the il-12rβ contained four FN3 domains and a transmembrane region. The ifnγ contained a conserved IFNγ family domain, and the ifnγr1 had a tissue factor domain and a transmembrane region. The csf1 consisted of a CSF-1 family domain and a transmembrane region, and the csf1r had a tyrosine protein kinases domain. Furthermore, the il-17a/f1, il-11, and il-34 had an IL-17 family domain, an IL-11 family domain and an IL-34 family domain, respectively (**Figure 5C**).

**Figure 6:**
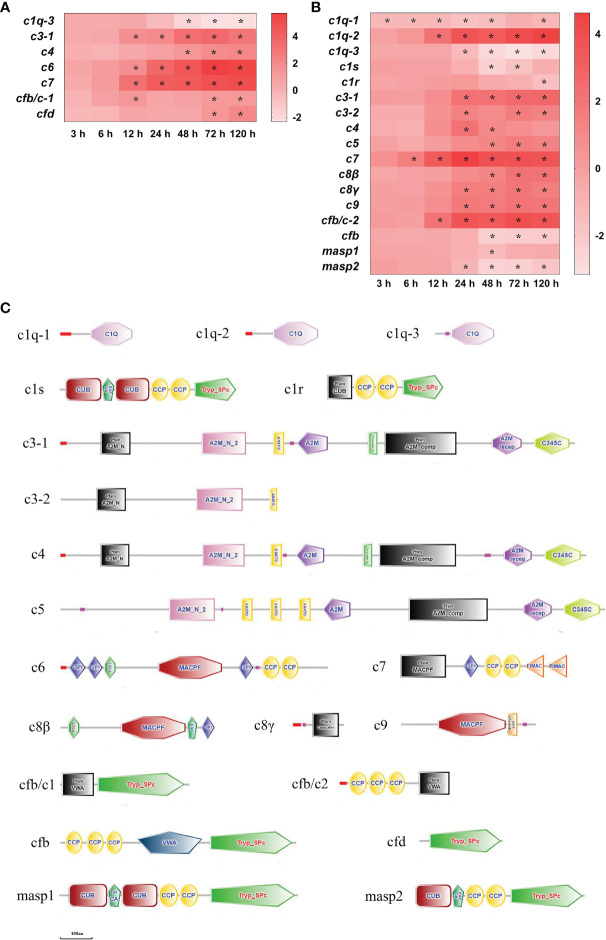
The DEGs of complement in yellow catfish after *E. ictaluri* infection. **(A**, **B)**: The DEGs identified in the spleen and liver, respectively. The DEGs of complement were analyzed at 3 h, 6 h, 12 h, 24 h, 48 h, 72 h, and 120 h post-infection in the spleen and liver. The color gradient represents highly up-regulated (red) to highly down-regulated (white) genes. Significant differences at different time points post-infection compared to the control (0 h) are indicated by asterisks (*P < 0.05). **(C)**: Structural features of complement DEGs in yellow catfish. The domain organizations were predicted by the SMART online server.

Altogether there were 19 complement (c) DEGs detected in the spleen and liver after infection (**Figure 6**). In the spleen, c3-1, c6 and c7 mRNAs were significantly up-regulated at 12–120 h (*P*<0.05), c4, complement factor (cf) b/c-1 and cfd mRNAs had high expression levels at two or three time points, respectively (*P*<0.05), however c1q-3 mRNA was notably down-regulated at 48–120 h (*P*<0.05) (**Figure 6A**). In the liver, c7 mRNA was notably up-regulated at 6–120 h (*P*<0.05), c1q-2 and cfb/c-2 mRNAs were significantly up-regulated at 12–120 h (*P*<0.05), c3-1, c8γ and c9 mRNAs were significantly up-regulated at 24–120 h (*P*<0.05), c3-2, c4, c5 and c8β mRNAs were notably up-regulated at two or three time points, respectively (*P*<0.05), whereas c1q-1mRNA was notably down-regulated at all time points except at 72 h (*P*<0.05), c1q-3 and masp2 mRNAs were significantly down-regulated at 24–120 h (*P*<0.05), cfb mRNA was significantly down-regulated at 48–120 h (*P*<0.05), and c1s, c1r and masp1mRNAs were notably down-regulated at one or two time points after 48 h of infection, respectively (*P*<0.05) (**Figure 6B**). The structure predictions of DEGs showed that three c1q homologous genes (c1q-1, c1q-2, c1q-3) contained a complement component C1Q domain, the c1r had a CUB domain, two complement control protein (CCP) domains and a trypsin-like serine protease (Tryp_SPc) domain, and the c1s consisted of two CUB domains, a calcium-binding EGF-like domain, two CCP domains and a Tryp_SPc domain (**Figure 6C**). The c3-1 and c4 comprised four alpha-2-Macroglobulin (A2M) domains, an A2M receptor (A2M recep) domain, an anaphylatoxin homologous (ANATO) domain and a netrin C-terminal (C345C) domain, the c5 contained three A2M domains, an A2M receptor domain, three ANATO domains and a C345C domain, and the c3-2 only had two A2M domains and an ANATO domain (**Figure 6C**). The c6, c7, c8β, and c9 all contained a membrane-attack complex perforin (MACPF), and the c8γ had a lipocalin domain. The cfb/c1, cfb, and cfd all had a trypsin-like serine protease (Tryp_SPc) domain, the cfb/c1, cfb/c2, and cfb all contained a von Willebrand type A (VWA) domain, and the cfb/c2 and cfb also comprised three CCP domains (**Figure 6C**). The masp1 consisted of two CUB domains, a calcium-binding EGF-like domain, two CCP domains and a Tryp_SPc domain, the masp2 was similar to the masp1, but it only had a CUB domain (**Figure 6C**).

**Figure 7:**
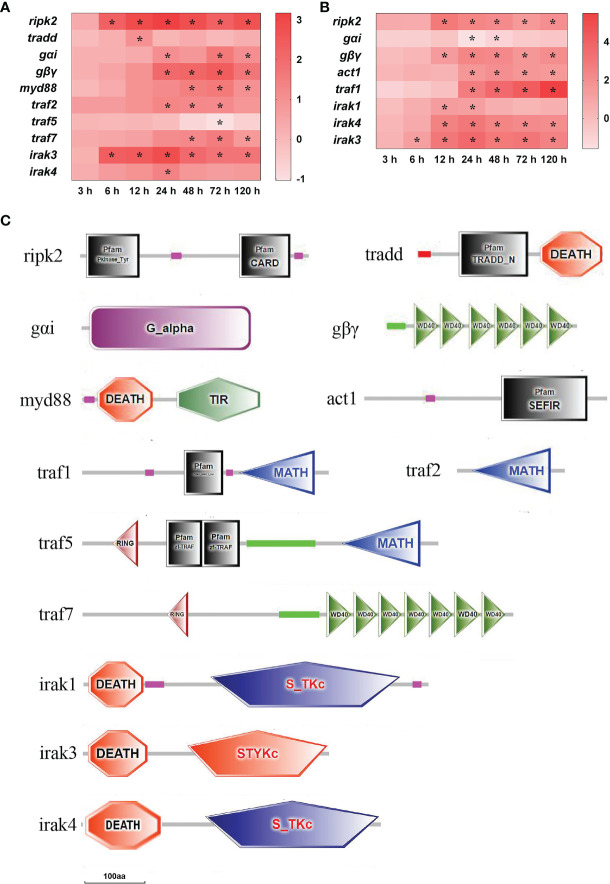
The DEGs of adaptor molecule in yellow catfish after *E. ictaluri* infection. **(A**, **B)**: The DEGs identified in the spleen and liver, respectively. The DEGs of adaptor molecule were analyzed at 3 h, 6 h, 12 h, 24 h, 48 h, 72 h, and 120 h post-infection in the spleen and liver. The color gradient represents highly up-regulated (red) to highly down-regulated (white) genes. Significant differences at different time points post-infection compared to the control (0 h) are indicated by asterisks (*P < 0.05). **(C)**: Structural features of adaptor molecule DEGs in yellow catfish. The domain organizations were predicted by the SMART online server.

In total, 13 important adaptor molecule DEGs were found in the spleen and liver after infection (**Figure 7**). In the spleen, ripk2 (receptor-interacting serine/threonine-protein kinase 2) mRNA was significantly up-regulated at 6–120 h, and tradd (tumor necrosis factor receptor type 1-associated DEATH domain protein) mRNA was notably up-regulated only at 12 h (*P*<0.05). The levels of gαi (guanine nucleotide-binding protein G(i) subunit alpha), gβγ (guanine nucleotide-binding protein subunit beta-5), myd88 (myeloid differentiation factor 88), traf2 (tumor necrosis factor receptor-associated factor 2) and traf7 mRNAs were notably up-regulated at three or four time points after 24 h of infection, respectively (*P*<0.05). The levels of irak3 (interleukin-1 receptor-associated factor 3) and irak4 mRNAs were significantly up-regulated at 6–120 h and at 24 h, respectively, while traf5 mRNA was distinctly down-regulated at 72 h (*P*<0.05) (**Figure 7A**). In the liver, the ripk2, gβγ, and irak4 mRNAs were significantly up-regulated at 12–120 h, act1 (adaptor protein CIKS) and traf1 mRNAs were notably up-regulated at 24–120 h, irak1, and irak3 mRNAs were significantly up-regulated at 12–24 h and at 3–120 h, respectively (*P*<0.05), whereas gαi mRNA was significantly down-regulated at 24–48 h (*P*<0.05) (**Figure 7B**). The structure predictions of DEGs showed that the ripk2 contained a protein tyrosine kinase domain and a caspase recruitment domain (CARD), and the tradd comprised a TRADD, N-terminal (TRADD_N) domain and a DEATH domain (**Figure 7C**). The gαi contained a G-protein alpha domain, and the gβγ had six WD domains. The myd88 consisted of a Death domain and a TIR domain, and the act1 had a SEFIR domain. The traf1, traf2, and traf5 all contained a meprin and TRAF homology (MATH) domain, besides, the traf1 had a TRAF_BIRC3_bd domain, the traf5 had a ring finger (RING) domain and two TRAF-type zinc finger (zf-TRAF) domains, and the traf7 consisted of a RING domain and seven WD domains (**Figure 7C**).The irak1, irak3, and irak4 all had a DEATH domain, while both the irak1 and irak4 also contained a serine/threonine protein kinases domain, and the irak3 also had an unclassified protein kinase domain (**Figure 7C**).

### Inflammation-Related Pathways of Yellow Catfish Against *E. ictaluri* Infection

Based on the 8,263 DEGs detected altogether in the spleen and liver, there were only ten pathways enriched significantly associated with the inflammation by KEGG enriched analysis (*P*<0.001). Among the ten pathways, more DEGs (122 and 117) were enriched into the complement and coagulation cascades (map04610) and the MAPK signaling pathway (map04010), whereas the JAK-STAT signaling pathway (map04630) and the TNF signaling pathway (map04668) were enriched with fewer DEGs (56 and 52), respectively (**Table 5**).

The MAPK signaling pathway is an important downstream signal transduction pathway of the inflammatory response, and it consists of ERK, JNK, and p38 signal pathways. In total, nine ERK signaling pathway-related DEGs were detected in the spleen and liver of yellow catfish after infection (**Supplemental Figure 1A**). In the spleen, GTPase HRas (hras), MAP kinase-interacting serine/threonine-protein kinase 1 (mknk1), activating transcription factor 4 (creb2) and proto-oncogene c-Fos-like (fos) mRNAs were distinctly up-regulated at four or five time points (*P*<0.05), son of sevenless homolog 1 (sos) and mitogen-activated protein kinase 1-like (erk) mRNAs were significantly up-regulated only at 72 h (*P*<0.05) (**Supplemental Figure 1A**). In the liver, GTPase NRas-like (kras) mRNA was notably up-regulated at 12–120 h (*P*<0.05), and RAF proto-oncogene serine/threonine-protein kinase-like (raf1) and activating transcription factor 4 (creb2) mRNAs were significantly up-regulated at 24 h (*P*<0.05), whereas transcriptional regulator Myc-B-like (myc) was significantly down-regulated at 3 h, and proto-oncogene c-Fos-like (fos) mRNA were notably down-regulated at all time points except at 12 h (*P*<0.05) (**Supplemental Figure 1A**). A total of 15 JNK and p38 signal pathway-related DEGs were found in the spleen and liver after infection (**Supplemental Figure 1B**). In the spleen, MAP kinase-activated protein kinase 3-like (mapkapk3), ras-related C3 botulinum toxin substrate 2 (rac2) and cell division control protein 42 homolog (cdc42) mRNAs were significantly up-regulated from 6 h or 12 h to 120 h (*P*<0.05), two mitogen-activated protein kinase 14A-like (p38-1 and p38-2) and mitogen-activated protein kinase kinase kinase 8 (cot) mRNAs were significantly induced at two or three time points (*P*<0.05), and mitogen-activated protein kinase kinase kinase 5 (ask1) was distinctly up-regulated only at 72 h (*P*<0.05); whereas mitogen-activated protein kinase kinase kinase kinase 2-like (gck) was notably down-regulated at 24–120 h (*P*<0.05), dual specificity mitogen-activated protein kinase kinase 4-like (mkk4) and transcription factor AP-1 (ap-1) mRNAs were notably down-regulated only at 72 h and 48 h, respectively (*P*<0.05) (**Supplemental Figure 1B**). In the liver, cot was significantly up-regulated at 6–120 h, cdc42 and p38-3 mRNAs were notably up-regulated at three or four time points, and mitogen-activated protein kinase kinase kinase kinase 4-like (hgk) was significantly up-regulated only at 24 h; whereas ap-1 and transcription factor jun-D-like (jund) mRNAs were significantly down-regulated at all time points (*P*<0.05), and ras-related C3 botulinum toxin substrate (rac1) mRNA was significantly down-regulated at 48–120 h (*P*<0.05) (**Supplemental Figure 1B**).

The JAK-STAT signal pathway mediates the downstream signal transduction pathway of IFN-γ and chemokine. Nine JAK-STAT signal pathway-related DEGs were detected in the spleen and liver after infection (**Supplemental Figure 1B**). In the spleen, suppressor of cytokine signaling 3 (socs3) mRNA was significantly up-regulated at all time points (*P*<0.05), signal transducer and activator of transcription 3 (stat3), signal transducer and activator of transcription 1 (stat1), suppressor of cytokine signaling 1 (socs1), tyrosine-protein kinase JAK2 (jak2) and signal transducer and activator of transcription 2 (stat2) mRNAs were notably induced from 12 h (or 24 h, 48 h) to 120 h (*P*<0.05), whereas signal transducer and activator of transcription 4 (stat4) mRNA was significantly down-regulated at 24–120 h (*P*<0.05) (**Supplemental Figure 1C**). In the liver, tyrosine-protein kinase JAK1 (jak1), stat1, jak2, and stat3 mRNAs were notably up-regulated from 6 h (or 12 h, 24 h) to 120 h (*P*<0.05), whereas stat2 mRNA was significantly down-regulated at 24–72 h (*P*<0.05) (**Supplemental Figure 1C**).

Additionally, there were seven other DEGs of inflammatory signal pathway detected in the spleen and liver after infection (**Supplemental Figure 1D**). In the spleen, caspase 3b (casp3) and NF-kappa-B inhibitor alpha-like (ikbα) mRNAs were significantly up-regulated from 3 h or 6 h to 120 h (*P*<0.05), inhibitor of nuclear factor kappa-B kinase subunit alpha (ikkα), NF-kappa-B inhibitor beta (ikbβ) and caspase 7 (casp7) mRNAs were notably induced at two or three time points, respectively (*P*<0.05), whereas caspase 1 (casp1) mRNA was significantly down-regulated at 24–120 h (*P*<0.05) (**Supplemental Figure 1D**). In the liver, ikbα, ikbβ and caspase 8 (casp8) mRNAs were notably up-regulated from 6 h (or 12 h, 48 h) to 120 h (*P*<0.05), respectively. The casp3 mRNA was significantly up-regulated only at 120 h (*P*<0.05) (**Supplemental Figure 1D**).

### qRT-PCR Analysis

**Figure 8:**
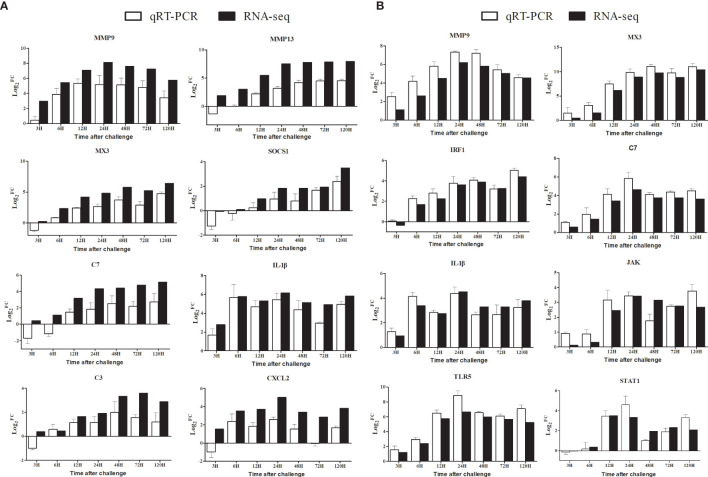
Comparison of the expression profiles of eight DEGs determined by Illumina HiSeq™ 2000 sequencing platform and qRT-PCR at 3 h, 6 h, 12 h, 24 h, 48 h, 72 h, and 120 h post-infection. Data shown are the mean of triplicates ± SD. **(A)**: Spleen, **(B)**: Liver.

We used qRT-PCR to validate RNA-seq expression profiles of nine selected genes that were highly expressed in the spleen and liver, respectively. As shown in **Figure 8**, despite some quantitative differences at the expression levels between the qRT-PCR and the RNA-seq, the qRT-PCR results revealed a similar expression tendency as the data of transcriptome, confirming the accuracy of the expression profiles in RNA-seq analyses.

## Discussion

Though water environment harbors a mass of pathogens, healthy fish can maintain the balance between host and pathogens by mucosal barrier including digestive enzymes, antimicrobial peptides and immunoglobulins (36, 37). In aquaculture, the deterioration of the water environment can lower fish immunity, and this will weaken mucosal barrier of fish and lead to the occurrence of fish diseases. Therefore, it is very essential to study immune-related gene functions and responses of fish against bacterial infection. In mammals, inflammation is a protective response by inducing large amounts of cytokine and complement genes to activate immune cells and eliminate pathogens (28). In fish, so far few research understands the inflammatory mechanisms underlying the bacteria-infection process.

### Dynamic Immune Process of Yellow Catfish Against *E. ictaluri*

In fish, many studies have been reported recently about transcriptome analysis of the immune response against bacterial infection for a certain tissue or at a certain time point post-infection, but there have been limited researches on the dynamic immune response against bacterial infection in time and space by transcriptome analysis. In this study, we used the RNA-Seq technology to study the dynamic immune response against *E. ictaluri* in the spleen and liver of yellow catfish at seven different time points post-infection. Here, we identified a total of 5,540 DEGs in the spleen and 4,161 DEGs in the liver of yellow catfish (**Table 4**). Overall, the number of annotated DEGs based on the GO database (>60%) was higher than that from the KEGG database (~20%) in the spleen and liver (**Table 5**), and this difference in yellow catfish was similar to those found in blunt snout bream (14), tilapia (38), topmouth culter (20) and mandarin fish (21). The GO and KEGG enrichment analyses of DEGs at different time points post-infection indicated the variability of biological functions in the spleen and liver of yellow catfish.

In the spleen of yellow catfish, the number of DEGs in the transcriptome was small (156 and 533) at 3 h and 6 h post-infection, respectively, subsequently it increased quickly to reach a peak value (4, 260) at 72 h. Cytokines are involved in every facet of immunity and inflammation (28). Chemokines are a group of small molecules that can induce chemotaxis in immune cells and regulate the activity of the immune cells through interactions with members of the 7-transmembrane, G-protein-coupled receptor superfamily (39). In the spleen of yellow catfish, cytokines and chemokines associated ontologies were found at 6–120 h, and the cytokine-cytokine receptor interaction (map04060) and the chemokine signaling pathway (map04062) were detected at 12–120 h in the KEGG enrichment analyses. G-protein-coupled receptors are the largest family of membrane proteins and mediate most cellular responses to hormones, neurotransmitters, ions, photons and other stimuli (40). In this study, G-protein-coupled-receptor associated GOs were discovered in the spleen at 12 h and 24 h in the GO enrichment analyses. These results imply that cytokines and chemokines can be continuously and distinctly transcribed in the spleen of yellow catfish from 6 h to 120 h after *E. ictaluri* infection.

In the liver of yellow catfish, the number of DEGs was also small (399 and 448) at 3 h and 6 h post-infection, respectively, but it maintained a high level (2,908, 3,148, 3,082, and 2,998) from 24 h to 120 h. IL-17 and TNF are strong pro-inflammatory cytokines in mammals (41, 42). In the current study, the KEGG enrichment analyses in the liver showed that the IL-17 signaling pathway (map04657), the TNF signaling pathway (map04668) and the Th17 cell differentiation (map04659) were only detected at 6 h or 12 h, and the complement and coagulation cascades (map04610) enriched the most DEGs at 24–120 h. These results suggest that the immune response in the liver may be activated at 6 h and 12 h post-infection by producing cytokines, afterwards the complement system might play a crucial role in immune response from 24 h to 120 h in the liver. During human infection, food intake will decrease with a change in leptin synthesis, and this will probably reduce the ingestion of other pathogens, activate energy-requiring mechanisms and diminish the competition of epitopes from nutrients for crucial receptors for pathogen sensing (43). In the liver of yellow catfish, metabolic and reproductive process associated GOs were detected at 12–120 h in the GO enrichment analyses, the fat digestion and absorption (map04975) was found at 12–120 h, the PPAR signaling pathway (map03320) and the glycerolipid metabolism (map00561) were found at 24–72 h in the KEGG enrichment analyses, indicating that the metabolic regulation in the liver can be activated at about 12 h post-infection to provide energy for yellow catfish against bacterial infection.

### Expression Changes and Potential Roles of Inflammation-Related DEGs Against *E. ictaluri*

**Figure 9:**
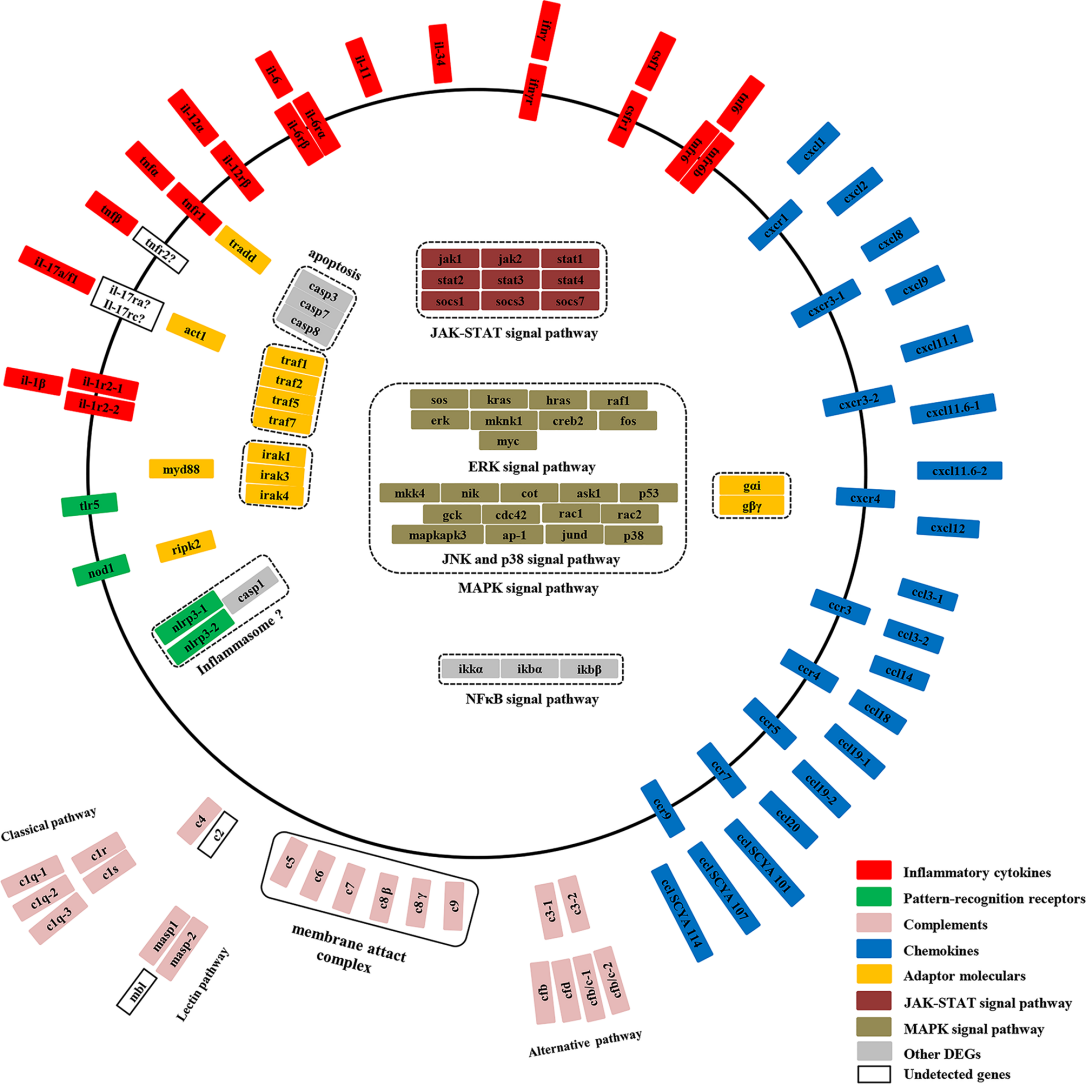
A graphic overview of the inflammation response of yellow catfish.

To better understand the inflammation response of yellow catfish against bacterial infection, a graphic overview of its inflammation response was drawn based on the transcriptome analysis (**Figure 9**). The pattern-recognition receptors can recognize specific components of microorganisms and trigger inflammatory responses, thus eliminate the invading microorganisms (44). In mammals, TLR5 recognizes the flagellin of bacterial flagella through MYD88 signaling (45), and NOD1 sense bacterial PGN fragments by RIPK2 signaling (46). In fish, two tlr5 genes (tlr5M and tlr5S) and nod1 mRNAs were up-regulated notably after bacterial infection (47, 48). In Japanese flounder, tlr5 can induce the expression of interleukin-1β and NF-κB genes (49). In yellow catfish, both tlr5M and nod1 mRNAs were induced notably and continuously in the liver from 6 h or 12 h to 120 h post-infection, and the predicted protein structures of two genes were similar to those of mammals. In mammals, NLRP3 can induce pro-inflammatory cytokines through recruiting apoptosis-associated speck-like protein (ASC) and Caspase-1 and constituting a multi-protein inflammasome complex (46). Japanese flounder nlrp3 mRNA is significantly up-regulated after bacterial infection (50). In yellow catfish, two nlrp3 genes (nlrp3-1 and nlrp3-2) were distinctly induced at 120 h and at 24–120 h, respectively. Moreover, compared with human NLRP3, two nlrp3 genes of yellow catfish lacked a PYRIN domain with important roles in binding to ASC (46), therefore it is necessary to further verify the function of yellow catfish nlrp3s in the future.

Once inflammatory processes are triggered, large amounts of pro-inflammatory cytokines are activated and released (28). In mammals, IL-1β and TNF, as symbolic pro-inflammation cytokines, can activate macrophages and neutrophils directly, and CSF-1 and IL-34 can drive monocyte differentiation into macrophages (41, 51, 52). IL-12 can promote the differentiation of type 1 helper T cells (Th1) (53). IFN-γ, derived from Th1 cells, can activate neutrophils (28). IL-17A and IL-17 F, derived mainly from Th17 cells, can induce the expressions of inflammatory cytokines, chemokines and antibacterial peptides (42). In fishes, multiple homologous genes of il-1β, TNFα, il-6, il-11, il-12α, il-17a/f and ifn-γ have been identified due to several whole-genome duplications in fish evolution, and they have similar bioactivity to those of mammals (54–58). In this study, yellow catfish il-1β, tnfα, tnf6, il-6, il-11, il-12α, il-17a/f1, and ifn-γ mRNAs were up-regulated significantly and continuously, while csf-1 and il-34 mRNAs were distinctly induced at late time points after *E. ictaluri* infection. Moreover, the predicted protein structures of these inflammatory cytokines contained their characteristic family domains. These results imply that yellow catfish immune cells can be activated quickly to eliminate invading pathogens.

In mammals, IL-1β can bind to IL-1R1 to activate MyD88 signaling, and IL-1R2 lacking the TIR domain can bind fraudulently to IL-1β and prevent signal transduction (59). TNFα can bind to TNFR1 and TNFR2 to activate TRADD signaling by a cytoplasmic DEATH domain (41). IFN-γ can activate the JAK-STAT signal pathway through IFN-γR1 and IFN-γR2 (60). In various fishes, il-1r1, il-1r2, tnfr1, tnfr2, ifnγr1 and ifnγr2 have been identified, and their mRNAs are notably induced after bacteria or LPS stimulation (60–64). In yellow catfish, il-1r1 and tnfr1 mRNAs were significantly up-regulated at 6–120 h post-infection, and ifnγr1 mRNA was notably induced at 48 h, 72 h, and 120 h, whereas il-1r2 mRNA was distinctly induced only at 3 h. Similar to mammals, yellow catfish il-1rs possess characteristic IG domains in the extracellular region and a cytoplasmic TIR domain except that il-1r2 lacks the TIR domain for signaling, and yellow catfish tnfr1 also contain a DEATH domain. In mammals, IL-6Rβ is a shared receptor in the IL-6 family, and IL-6 and IL-11 also need to bind to IL-6Rα and IL-11R, respectively, to activate the JAK-STAT signal pathway (65). IL-12α can bind to IL-12/IL-23β, and then combine with IL-12Rβ1 and IL-12Rβ2 to activate the JAK-STAT signal pathway (53). So far, little is known about fish il-6rα, il-6rβ and il-12rβ. Rainbow trout il-6rα and il-6rβ have been identified and their mRNAs are up-regulated after stimulation with LPS and poly I:C (66). In this study, yellow catfish il-6rα and il-6rβ mRNAs were significantly up-regulated from 3 h or 12 h to 120 h post-infection. Yellow catfish il-12rβ possesses four fibronectin type III domains, while its mRNA was notably down-regulated at 48 h, 72 h, and 120 h. These results reveal that compared with mammals, inflammatory cytokine receptors in fish have similar protein structure and expression changes after bacterial infection, and the signal pathways of these inflammatory cytokines are conserved.

Chemokines play a crucial role in inflammation and immunity (39). In humans, 44 chemokines and 23 chemokine receptors have been identified (67). In teleosts, the family of chemokine is more complex than that in mammals. More than 100 different chemokines have been found in zebrafish (68). Only a few orthologues of mammalian chemokines have been identified in fish, such as cxcl8, cxcl11, cxcl12, ccl19 and ccl20. There is a limited understanding of the bioactivity of fish chemokines in the immune response. In mammals, CXCL1–CXCL3 and CXCL5–CXCL8 have the conserved “ELR” motif, and they are chemotactic factors for neutrophils, while CXCL9–CXCL12 lacking “ELR” motif can recruit and activate lymphocytes T and NK cells (69). Mammalian CC chemokines are chemoattractant for macrophages and neutrophils (70). In this study, yellow catfish cxcls and ccls also have a typical C-X-C and C-C structure as mammals, respectively. Moreover, cxcl1, cxcl2, cxcl9, cxcl11.1, cxcl11.6-1, cxcl11.6-2, cxcl12, ccl3-2, ccl19-1, ccl19-2, ccl20, and cclSCYA 101, 107, 114 mRNAs were significantly and continuously induced after *E. ictaluri* infection, implying that yellow catfish immune cells can be activated and recruited to the sites of infection at early time points post-infection. In mammals, chemokine signals are transduced through binding to members of the seven-transmembrane, G-protein-coupled receptor superfamily (29). In yellow catfish, multiple genes of cxcrs and ccrs were detected to have a seven-transmembrane domain. Furthermore, cxcr1, cxcr3-2, ccr3, and ccr5 mRNAs were notably up-regulated at 6 h or 12 h and maintained until 120 h post-infection, whereas cxcr3-1, cxcr4, ccr4, ccr7, and ccr9 mRNAs were notably down-regulated after *E. ictaluri* infection, therefore it is necessary to further verify the bioactivity of these chemokine receptors of yellow catfish in the following study. Besides, CCL14 and CCL18 can inhibit the pro-inflammatory immune response (71, 72). Similarly, yellow catfish ccl14 and ccl18 mRNAs were down-regulated after *E. ictaluri* infection, suggesting that the ccl14 and ccl18 may play crucial anti-inflammation roles in fish.

Complement has been viewed as a supportive “first line of defense” against microbial intruders, and can quickly tag and eliminate them (73). A variety of complement genes have been identified in fish (74), but the function and signaling pathway of fish complement are not clear. There are three complement pathways in mammals: classical, alternative and lectin (73). The classical pathway is activated by C1 family (C1q, C1r, and C1s) cleaving C4 and C2. In contrast, the lectin pathway is activated through mannose-binding lectin (MBL) and MASPs cleaving both C4 and C2 (73). After *E. ictaluri* infection in yellow catfish, three C1q genes (c1q-1, c1q-2, and c1q-3) with a C1Q domain were detected and only c1q-2 was notably induced in the liver, whereas c1r and c1s were notably down-regulated in the liver. Yellow catfish mbl was not detected and two masp genes (masp1 and masp2) were notably down-regulated in the liver after bacterial infection. Yellow catfish c2 was not detected, and c4 was distinctly induced in the spleen and liver after bacterial infection. Similar to mammals, two masp genes of yellow catfish share structural similarity with c1r and c1s. In the alternative pathway of mammals, C3 is cleaved by the factor B (FB) and factor D (FD) to activate complement (73). In yellow catfish, two c3 genes were detected to be induced significantly after *E. ictaluri* infection. Meanwhile, cfb was notably down-regulated, whereas two cfb/c genes (cfb/c-1 and cfb/c-2) with similar protein structure to cfb were significantly up-regulated, so did cfd gene. These results imply that yellow catfish has complement activation pathways similar to mammals, the alternative pathway of yellow catfish may be activated after *E. ictaluri* infection, but the classical and lectin pathways may be suppressed. While the complement pathways are activated, C5 is cleaved to the anaphylatoxin C5a and fragment C5b, and then C5b, C6, C7, C8 and C9 can form MAC to eliminate pathogen (73). In yellow catfish, c5–c9 mRNAs had high expression levels in the spleen or liver, and c6–c9 contained a MAC domain, implying that they might play potential roles in the immune responses of yellow catfish against pathogens.

In mammals, MyD88 is an important adaptor molecule involved in the IL-1/TLR signaling pathway (51). TRADD, as an important adaptor molecule of TNF signal pathway, can combine with TNFR to activate the downstream signaling (41). NOD1 and NOD2 can activate NF-κB pathway through CARD domain recruiting adaptor molecule RIPK-2 (75). ACT1 can bind to IL-17R to modulate IL-17 signaling (42). In various fish, myd88 has been detected to have high transcripts levels after pathogens infection, while little is known for fish tradd, ripk-2 and act1 genes. In this study, yellow catfish myd88, tradd, ripk-2, and act1 mRNAs were significantly induced after *E. ictaluri* infection, and four genes possessed similar protein structures to those of mammals. In mammals, seven TRAF proteins have emerged as adapter proteins to control the signal transduction of IL-1, IL-17, and TLR family (76). TRAF proteins consist of a unique and highly conserved MATH domain, but the C-terminus of TRAF7 is replaced by seven WD40 repeats (76). In teleosts, little is known about the traf gene family except that fish traf6 might be involved in the innate immune response (77). In the present study, yellow catfish traf1, 2, 5 and 7 were detected to have conserved protein structures, and traf1, 2 and 7 mRNAs were notably up-regulated after *E. ictaluri* infection. In mammals, IRAK proteins are an important class of kinases involved in the IL-1/TLR signaling pathway, and all four IRAKs have similar protein architectures: an N-terminal death domain and a kinase domain (78). So far there are few studies on IRAKs from teleosts. Recently, some reports suggest that fish IRAK4 could suppress MyD88-dependent NF-kB activation (77, 79). In this study, yellow catfish irak1, 3, and 4 mRNAs were distinctly induced after *E. ictaluri* infection and the three iraks genes had a death domain and a kinase domain as mammals. These results exhibit that these adaptor molecules have conserved protein structures, but their bioactivity need to further be explored in fish.

In conclusion, this study showed the transcriptomic profiles of yellow catfish in the spleen and liver at 3 h, 6 h, 12 h, 24 h, 48 h, 72 h, and 120 h after *E. ictaluri* infection, evidencing that an extensive transcriptional change was elicited at 12–120 h post-infection in the spleen and liver. Subsequently, the GO and KEGG enrichment analyses of DEGs were performed to uncover the dynamic immune responses of yellow catfish in the spleen and liver at different time points post-infection. On the whole, inflammatory cytokines and chemokines mRNAs were quickly and distinctly up-regulated at early time points (3 or 6 h) post-infection, then complement component mRNAs were induced continuously and notably from 12 or 24 h. In terms of mRNA tissue expressions, inflammatory cytokines and chemokines were mainly produced in the spleen, while more complement genes were induced in the liver after *E. ictaluri* infection. Finally, the inflammation response of yellow catfish was graphically overviewed and discussed, including pattern-recognition receptors, inflammatory cytokines, chemokines, complements and inflammation-related signal pathways. Overall, the transcriptomic data and relevant analyses will help to better understand the molecular defense mechanisms against bacterial infection, and will provide basic information to further study the immune regulation of bacterial diseases in yellow catfish.

## Data Availability Statement

The datasets presented in this study can be found in online repositories. The transcriptome data have been deposited to NCBI (BioProject ID: PRJNA690649), and the accession numbers are SRR13385414 to SRR13385461.

## Ethics Statement

The animal study was reviewed and approved by Institutional Animal Care and Use Committees (IACUC) of HZAU, Wuhan, P. R. China.

## Author Contributions

XZ performed the experiments, analyzed the data, and wrote the manuscript. XZ, G-RZ, WJ, X-FM, Z-LL and K-JW conceived and designed the study. Z-CS helped with the preparation of experimental fishes. XZ, Z-LL and K-JW revised the manuscript. All authors contributed to the article and approved the submitted version.

## Funding

This study was supported by the Biodiversity Survey and Assessment Project of the Ministry of Ecology and Environment, China (Grant No. 2019HJ2096001006), and the National Natural Science Foundation of China (Grant No. 31772851).

## Conflict of Interest

The authors declare that the research was conducted in the absence of any commercial or financial relationships that could be construed as a potential conflict of interest.
