# Supplementary material for: The Dynamic Immune Response of Yellow Catfish (Pelteobagrus fulvidraco) Infected With Edwardsiella ictaluri Presenting the Inflammation Process
Source: Front Immunol. 2021 Feb 26;12:625928. doi: 10.3389/fimmu.2021.625928 (PMC7959794; doi:10.3389/fimmu.2021.625928)

Supplementary material

Supplementary Table 1. Primers used for qRT-PCR in this study

Primer name	Sequence (5'-3')	Ta (°C)	Application
MMP9-QF1	CAAGGGACCCGCAATCAA	64	qRT-PCR of MMP9 mRNA
MMP9-QR1	CTCAAACACAAACGGAAAGACAC		
MMP13-QF1	GAGACGCACATTTTGATGACGA	64	qRT-PCR of MMP13 mRNA
MMP13-QR1	GCATTAGGGGTGACTGGAGGT		
IRF1- QF1	CCTGGTTAGAGGATATGATTG	62	qRT-PCR of IRF1 mRNA
IRF1- QR1	TGGCTTGAACTTGCCTGTGTG		
JAK1- QF1	CTGACCAAGAGCATCAAAGACA	62	qRT-PCR of JAK1 mRNA
JAK1- QR1	TGACTCCGAAAGACCAGACAT		
MX3- QF1	TGTGACCTCACATTGATCG	64	qRT-PCR of MX3 mRNA
MX3- QR1	ATTGCCAGAGTCCTTTTTC		
CXCL2- QF5	ATCTGCTGACCATTGCCCTT	62	qRT-PCR of CXCL2 mRNA
CXCL2- QR5	GTCCTGACCTTCATCACCTT		
TLR5S- QF7	AGATGATAAAGCCTACTGTGCC	62	qRT-PCR of TLR5S mRNA
TLR5S- QR7	GTTGAATAATCCGTTGAATG		
IL-1β- QF1	TCTCAGCCTACAACCCACCA	64	qRT-PCR of IL-1βmRNA
IL-1β- QR1	CTCCATTCCATCGTTCTCCT		
C7- QF1	TTCCGAAAGAAGAGAACGAC	62	qRT-PCR of C7 mRNA
C7- QR1	ATTTGGCATACAGGGCATAG		
C3-QF1	GTGGTCTTGGTATCGTTTCA	62	qRT-PCR of C3 mRNA
C3-QR1	GTTCTTCTGTGGGGTGTTCT		
STAT1- QF1	GTTTTGCAATGGAGAAGGACTT	62	qRT-PCR of STAT1 mRNA
STAT1- QR1	CGCTCTGGGCGTTATGTG		
SOCS1- QF1	AAACCGCACGCACTTCCGC	62	qRT-PCR of SOCS1 mRNA
SOCS1- QR1	ACGACTCCTTGCTTCCCATAA		

Supplementary Table 2. Quality of clean reads.

Liver sample	Raw reads	Clean reads	Error rate (%)	Q20 (%)	Q30 (%)	GC content (%)	Spleen sample	Raw reads	Clean reads	Error rate (%)	Q20 (%)	Q30 (%)	GC content (%)
L0_1	54936058	54583448	0.0231	98.86	96.06	48.01	SP0_1	45380016	44937534	0.0260	97.77	93.03	46.16
L0_2	43228912	42898104	0.0235	98.72	95.65	48.49	SP0_2	45850978	45121260	0.0242	98.44	94.89	45.99
L0_3	46031806	45678528	0.0232	98.81	95.90	48.21	SP0_3	45583062	45122650	0.0242	98.42	94.87	45.71
L1_1	52607134	52237092	0.0232	98.81	95.92	48.2	SP1_1	48533786	48014096	0.0235	98.70	95.56	47.16
L1_2	55406810	54989626	0.0231	98.87	96.08	47.91	SP1_2	53383662	52765646	0.0236	98.67	95.52	46.78
L1_3	75135220	74602134	0.0232	98.81	95.89	47.84	SP1_3	49168408	48621156	0.0240	98.51	95.09	46.10
L2_1	41583944	41183944	0.0232	98.79	95.91	48.55	SP2_1	49919934	49248870	0.0237	98.63	95.39	46.16
L2_2	57448692	56906210	0.0237	98.66	95.32	48.22	SP2_2	44062228	43442090	0.0239	98.53	95.17	45.29
L2_3	52921372	52498140	0.0235	98.76	95.60	48.29	SP2_3	48035714	47492188	0.0239	98.55	95.17	46.69
L3_1	55483338	54979586	0.0234	98.76	95.62	47.85	SP3_1	55864424	55154068	0.0244	98.34	94.62	46.38
L3_2	48944908	48504310	0.0237	98.66	95.32	47.97	SP3_2	54005782	53394024	0.0237	98.62	95.36	46.07
L3_3	49276672	48849826	0.0232	98.87	95.90	48.34	SP3_3	53100862	52495740	0.0261	97.72	92.98	46.30
L4_1	41039960	40687480	0.0238	98.65	95.26	48.19	SP4_1	57916960	57271322	0.0240	98.51	95.07	50.42
L4_2	58398948	57905246	0.0236	98.7	95.43	47.95	SP4_2	54085020	53451438	0.0240	98.48	95.05	52.67
L4_3	57317136	56889134	0.0234	98.79	95.68	48.25	SP4_3	53750388	53207732	0.0241	98.50	94.98	47.85

Liver sample	Raw reads	Clean reads	Error rate (%)	Q20 (%)	Q30 (%)	GC content (%)	Spleen sample	Raw reads	Clean reads	Error rate (%)	Q20 (%)	Q30 (%)	GC content (%)
L5_1	59029490	58573816	0.0236	98.71	95.48	47.81	SP5_1	50416070	49743444	0.0243	98.39	94.71	45.02
L5_2	56526354	56052566	0.0232	98.85	95.87	47.94	SP5_2	62082818	61547214	0.0237	98.63	95.35	48.27
L5_3	68236198	67656714	0.0232	98.85	95.89	48.12	SP5_3	52141374	51661622	0.0238	98.61	95.29	47.08
L6_1	58687178	58152868	0.0234	98.79	95.70	48.02	SP6_1	57689906	57111472	0.0238	98.58	95.20	48.05
L6_2	50884676	50433018	0.0232	98.85	95.87	48.29	SP6_2	61287914	60647154	0.0237	98.63	95.35	47.45
L6_3	51765446	51270840	0.0233	98.83	95.83	48.52	SP6_3	53832336	53339050	0.0239	98.54	95.09	47.40
L7_1	52949400	52439184	0.0237	98.65	95.31	48.42	SP7_1	53802982	53248664	0.0240	98.54	95.08	46.09
L7_2	52490710	51988918	0.0236	98.71	95.48	47.76	SP7_2	59436462	58899122	0.0241	98.50	94.98	47.18
L7_3	52183800	51735072	0.0232	98.85	95.86	47.74	SP7_3	54652912	54074396	0.0242	98.45	94.84	46.14

L0~7 and SP0~7 represented the samples of liver and spleen at 0, 3, 6, 12, 24, 48, 72, and 120 hours post-injection of *Edwardsiella ictaluri*, respectively.

Supplementary Table 3. Reads mapping information.

Liver sample	Clean reads	Mapped reads	Mapped ratio	Spleen sample	Clean reads	Mapped reads	Mapped ratio
L0_1	27291724	23111105	84.68%	SP0_1	22468767	16969545	75.53%
L0_2	21449052	18343816	85.52%	SP0_2	22560630	17660621	78.28%
L0_3	22839264	19319166	84.59%	SP0_3	22561325	16969116	75.21%
L1_1	26118546	22062834	84.47%	SP1_1	24007048	18983688	79.08%
L1_2	27494813	23199495	84.38%	SP1_2	26382823	20717482	78.53%
L1_3	37301067	31126022	83.45%	SP1_3	24310578	18421943	75.78%
L2_1	20591972	17318324	84.10%	SP2_1	24624435	19298143	78.37%
L2_2	28453105	23835813	83.77%	SP2_2	21721045	16799410	77.34%
L2_3	26249070	21915842	83.49%	SP2_3	23746094	18392729	77.46%
L3_1	27489793	22788829	82.90%	SP3_1	27577034	21283223	77.18%
L3_2	24252155	20197581	83.28%	SP3_2	26697012	20967169	78.54%
L3_3	24424913	20535620	84.08%	SP3_3	26247870	20119343	76.65%
L4_1	20343740	16875541	82.95%	SP4_1	28635661	22440976	78.37%
L4_2	28952623	24003207	82.91%	SP4_2	26725719	20949924	78.39%
L4_3	28444567	23715245	83.37%	SP4_3	26603866	21311881	80.11%
L5_1	29286908	24189032	82.59%	SP5_1	24871722	19134475	76.93%
L5_2	28026283	23365301	83.37%	SP5_2	30773607	24314454	79.01%
L5_3	33828357	28303215	83.67%	SP5_3	25830811	20350221	78.78%
L6_1	29076434	24053931	82.73%	SP6_1	28555736	22846447	80.01%
L6_2	25216509	20942811	83.05%	SP6_2	30323577	24056268	79.33%
L6_3	25635420	21285607	83.03%	SP6_3	26669525	20766431	77.87%
L7_1	26219592	21896347	83.51%	SP7_1	26624332	20806822	78.15%
L7_2	25994459	21682586	83.41%	SP7_2	29449561	22820880	77.49%
L7_3	25867536	21519169	83.19%	SP7_3	27037198	21018400	77.74%

L0~7 and SP0~7 represented the samples of liver and spleen at 0, 3, 6, 12, 24, 48, 72, and 120 hours post-injection of *E. ictaluri*, respectively.

Supplementary Table 4. Evaluation of transcriptome assembly results at all time points in the spleen and liver.

Transcripts numbers	Unigenes numbers	Sequence base (G)	Min Length (bp)	Max Length (bp)	N50 Length (bp)
481943	321257	379.34	201	19109	1368

Supplemental Figure 1. The inflammation signal pathway-related DEGs in the spleen and liver of yellow catfish after *E. ictaluri* infection. The DEGs of adaptor molecule were analyzed at 3 h, 6 h, 12 h, 24 h, 48 h, 72 h and 120 h post-infection in the spleen and liver. The colour gradient represents highly up-regulated (red) to highly down-regulated (white) genes. Significant differences at different time points post-infection compared to the control (0 h) are indicated by asterisks (*: $P < 0.05$).

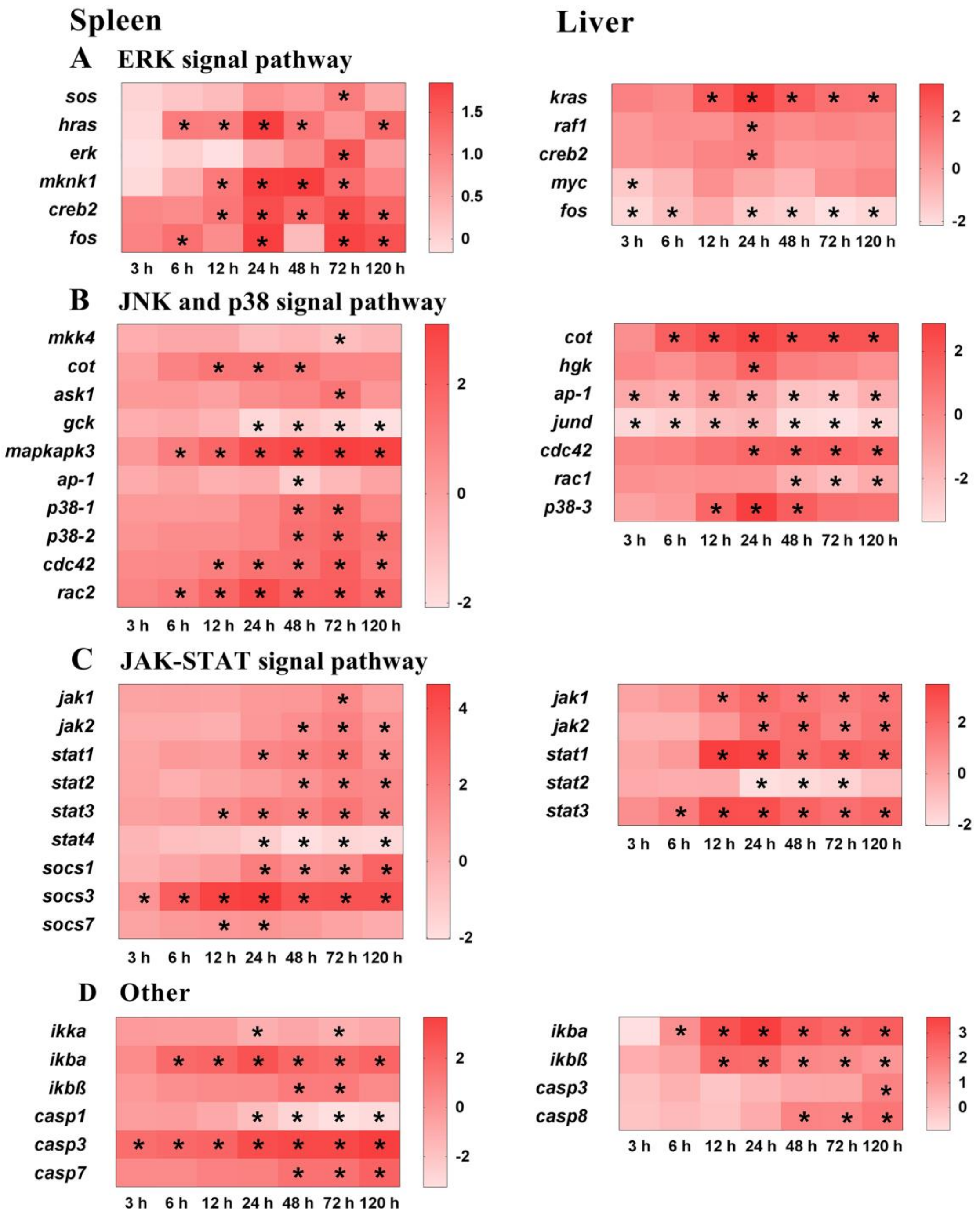

Supplement: Supplementary file 1 [file DataSheet_1.pdf]
